# First-trimester maternal serum alpha-fetoprotein is not a good predictor for adverse pregnancy outcomes: a retrospective study of 3325 cases

**DOI:** 10.1186/s12884-020-2789-2

**Published:** 2020-02-12

**Authors:** Jilin Hu, Jinman Zhang, Guilin He, Shu Zhu, Xinhua Tang, Jie Su, Qian Li, Yamin Kong, Baosheng Zhu

**Affiliations:** National Health Commission Key Laboratory of Periconception Health Birth in Western China, Yunnan Provincial Clinical Medicine Research Center for Birth Defects and Rare Diseases, Department of Obstetrics and Gynecology, the First People’s Hospital of Yunnan Province, No. 157, Jinbi Road, Xishan District, Kunming, Yunnan Province 650032 People’s Republic of China

**Keywords:** Alpha-fetoprotein, First-trimester, Preterm birth, Stillbirth, Preeclampsia, Small for gestational age

## Abstract

**Background:**

It is well known that second-trimester maternal serum alpha-fetoprotein (MS-AFP) is a predictor for adverse pregnancy outcomes (APOs), such as preterm birth, stillbirth, preeclampsia and small for gestational age (SGA). However, it is unknown whether first-trimester MS-AFP is also predictive of APOs.

**Methods:**

We retrospectively reviewed the data on the first-trimester MS-AFP levels and pregnancy outcomes of 3325 singleton pregnant women. The cutoff value of 2.5 multiple of the median (MoM) was used to evaluate the risks of APOs regarding MS-AFP. The receiver operating characteristic (ROC) curves were used to evaluate the predictive efficiencies of MS-AFP to these disorders.

**Results:**

A total of 181 pregnancies resulted in preterm birth, 32 in stillbirth, 81 in preeclampsia, and 362 in SGA. Compared to women with MS-AFP < 2.5MoM, those with MS-AFP ≥ 2.5MoM had increased risks (odds ratio, 95% confidence interval) of preterm birth (2.53, 1.65~3.88), preeclampsia (3.05, 1.71~5.43) and SGA (1.90, 1.34~2.69), and had an earlier distribution of gestational weeks at delivery (*P* = 0.004) and a lower distribution of neonatal birth weights (*P* = 0.000), but the actual between-group differences were minuscule. The areas under ROC curves were 0.572 (*P* = 0.001), 0.579 (*P* = 0.015) and 0.565 (*P* = 0.000) for preterm birth, preeclampsia and SGA, respectively. Subdivisions for the disorders did not obviously improve the performances of MS-AFP.

**Conclusions:**

Elevated first-trimester MS-AFP is associated with increased risk of preterm birth, preeclampsia and SGA. However, the predictive efficiencies were low and it is not a good predictor for these APOs.

## Introduction

Adverse pregnancy outcomes (APOs), such as preterm birth, stillbirth, preeclampsia and small for gestational age (SGA), are the major causes of fetal, neonatal and even maternal death and complications [1, 2]. In the recent years, much progress has been made in the first-trimester screening of preeclampsia and low-dose aspirin treatment to prevent the disease [3]. In 2018, low-dose aspirin was recommended by the American College of Obstetricians and Gynecologists (ACOG) as the prophylactic treatment for women at high risk of preeclampsia [4]. However, except for preeclampsia, other APOs lack effective methods for prediction so far.

Maternal serum alpha-fetoprotein (MS-AFP) is a second-trimester biochemical marker for prenatal screening. High or low MS-AFP suggests high risk of fetal open neural tube defects (ONTDs) or chromosomal aneuploidy, respectively [5]. Based on the decades’ experience of prenatal screening, it has been reported in numerous studies that after excluding fetal ONTDs, women with elevated second-trimester MS-AFP have higher risk of APOs [6–12]. Therefore, it has been well known that second-trimester MS-AFP can be a biomarker for the predictions of APOs.

In the recent years, the use of first-trimester screening has been considered as a window of opportunity to predict and prevent APOs [13]. Maternal serum analytes such as pregnancy-associated plasma protein-A (PAPP-A) and placental growth factor (PlGF), which have already been used in the screening of preeclampsia and been proven to be associated with APOs, were also available in the first trimester [14–16]. However, MS-AFP is routinely tested only in the second trimester, and it is unknown whether first-trimester MS-AFP can also be as predictive of APOs as second-trimester MS-AFP. Therefore, we aimed to investigate the predictive value of first-trimester MS-AFP to preterm birth, stillbirth, preeclampsia and SGA in this study.

## Materials and methods

### Study population

The study population was all of the 3427 women with singleton pregnancies who received first-trimester prenatal screening in the First People’s Hospital of Yunnan Province, China, from January to December 2016. The screening includes β-human chorionic gonadotropin (β-HCG) and PAPP-A tests, and Doppler ultrasound scan in 11~13^+ 6^ gestational weeks. The gestational age were determined by crown-rump length measured by ultrasound scan. In order to promote the first-trimester one-stop screening, we performed MS-AFP test additionally combining with ultrasonic measurement of intracranial translucency (IT) and detection of fetal structural abnormalities to study the first-trimester screening of fetal ONTDs. The study on ONTDs had not ended yet due to its very low incidence rate which was reported to be 0.045% by the Ministry of Health of P.R. China in 2012, but we retrospectively reviewed the data on the first-trimester MS-AFP levels and pregnancy outcomes of the 3427 women and investigated the association of first-trimester MS-AFP with APOs in this study.

### Measurement of MS-AFP

The serum samples of the study population were collected in the first-trimester one-stop screening. MS-AFP was measured using Time-resolved Fluorescence Immunoassay (TRFIA) kit (PerkinElmer Life and Analytical Sciences, Wallac, Turku, Finland) performed on AutoDELFIA1235 System. The measured values of MS-AFP were converted to the multiple of the median (MoM), referring to the gestational age-specific MS-AFP median values from our own database. 2.5 MoM was used as the cutoff value of MS-AFP, and was used to evaluate the risks of the APOs regarding MS-AFP.

### Study design

**Fig. 1:**
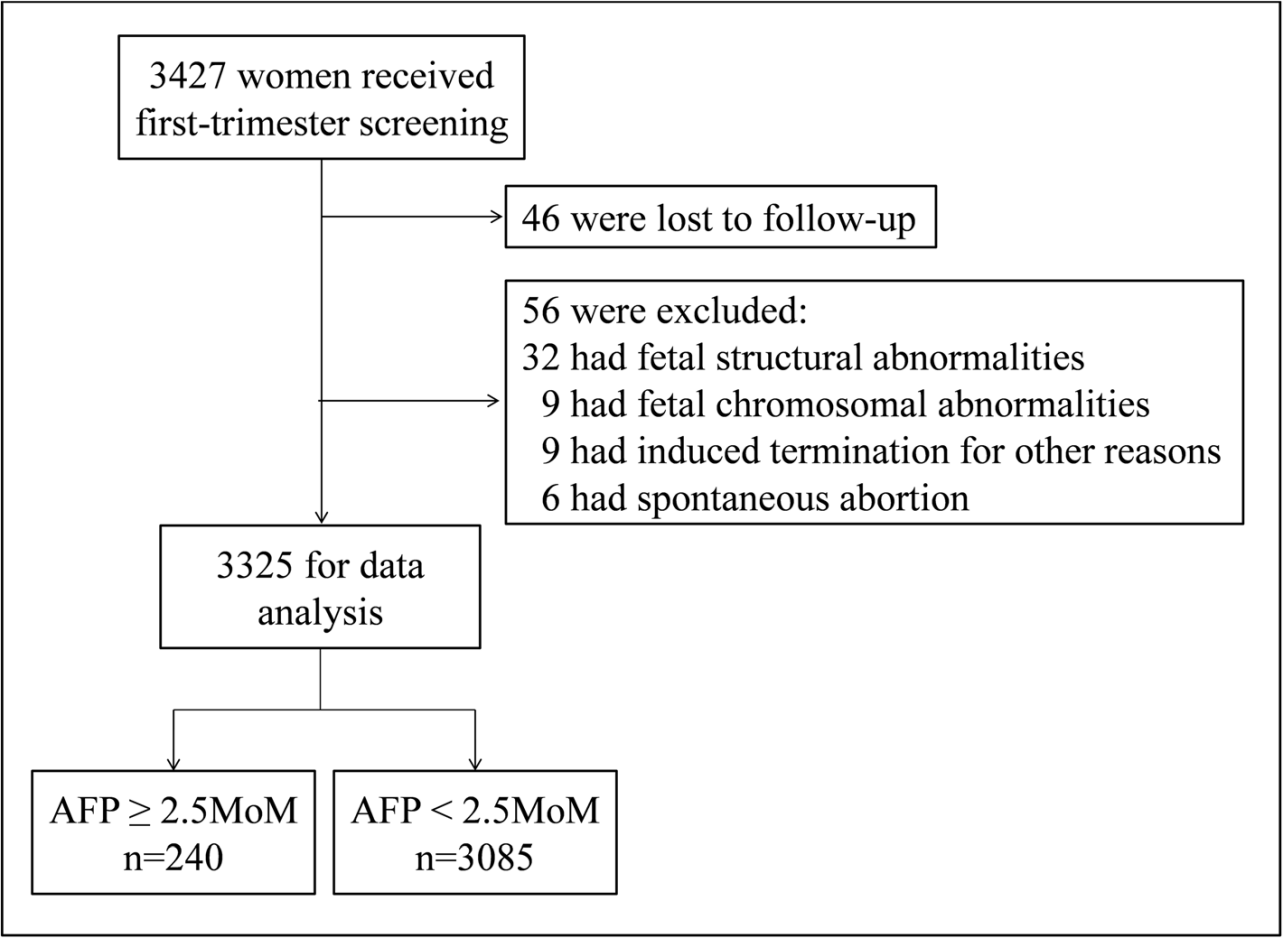
Participants selection flowchart and the number of participants among subgroups of the study population

Methods for participants selection was shown in Fig. 1. Among 3427 women, 46 of them were lost to follow-up, 50 were excluded because of fetal structural abnormalities (*n* = 32), fetal chromosomal abnormalities (*n* = 9) and induced termination of pregnancy for other reasons (*n* = 9). Six cases of spontaneous abortion were also excluded, because its low incidence was inadequate for analysis. Finally, the data of 3325 women were used for analysis. To confirm the pregnancy outcomes, all women were followed up by in-patient medical records review, supplemented by telephone follow-up. Preterm birth was defined as delivery between 20 and 37 gestational weeks [17]. Stillbirth was the intrauterine fetal death diagnosed by ultrasound scan or the delivery of a fetus showing no signs of life at 20 weeks or greater of gestation [18]. Preeclampsia was diagnosed by the new onset of maternal hypertension after 20 gestational weeks and the coexistence of proteinuria, or fetal growth restriction, or maternal organ dysfunction [19]. SGA was defined as a birth weight below the 10th percentile according to gestational age. The birth weight percentile was calculated using a contemporaneously described birth weights of local population [20]. Stillbirth and preeclampsia were looked at independently, while SGA and preterm birth were not. SGA was all the cases with live birth neonates’ weight below the 10th percentile. Preterm birth included all the cases who delivered between 20 and 37 gestational weeks.

### Statistical analysis

The results were analyzed statistically using IBM SPSS Statistics 19.0 (IBM Corp., Armonk, NY, USA). The descriptive statistics for continuous variables were expressed as “mean ± standard deviation” or “median (interquartile range)”, while categorical variables were expressed in the number and percentage. Student t test or Mann-Whitney test was used for continuous variables according to the data distribution, and Chi-square or Fisher’s exact test for categorical variables according to the expected counts. The odds ratio (OR) and 95% confidence interval (CI) were calculated to evaluate the relative risk of APOs regarding MS-AFP. The receiver operating characteristic (ROC) curves were used to evaluate the predictive efficiency. A Kaplan-Meier curve was used to show the overall distribution of gestational weeks at delivery. *P* < 0.05 was considered statistically significant.

## Results

Among 3325 women enrolled in the study, 240 (7.22%) of them had MS-AFP ≥ 2.5 MoM, and 3085 (92.78%) had MS-AFP < 2.5 MoM. Participants’ basic characteristics such as maternal age, weight, body mass index, parity, smoking history, gestational diabetes mellitus, neonatal gender and delivery mode, and screening markers β-HCG and nuchal translucency (NT) were all not significantly different between women with MS-AFP ≥ 2.5 and < 2.5 MoM. However, women with elevated MS-AFP had lower PAPP-A levels (*P* = 0.001) and higher proportion of assisted reproductive technology (*P* = 0.003) (Table 1).

**Table 1:** Participants’ characteristics and screening markers

	MS-AFP ≥ 2.5 MoM	MS-AFP < 2.5 MoM	*P* value
Maternal age (years)	30.55 ± 4.43	30.99 ± 4.36	0.139
Maternal age ≥ 35 years (n, %)	35 (14.58)	571 (18.51)	0.129
Maternal weight (kg)	55.65 ± 9.31	55.63 ± 8.59	0.980
Maternal height (cm)	160.09 ± 5.10	160.40 ± 4.80	0.353
Body mass index	21.66 ± 3.44	21.61 ± 3.12	0.823
Neonatal gender (male/female)	114/126	1546/1439	0.435
Parity (2/1/0)	68/78/94	755/1028/1302	0.392
Smoking history (n, %)	0 (0.00)	8 (0.26)	1.000
Gestational diabetes mellitus (n, %)	26 (10.83)	293 (9.50)	0.499
Assisted reproductive technology (n, %)	14 (5.83)	78 (2.53)	0.003
Caesarean section (n, %)	104 (43.33)	1264 (40.97)	0.474
Forceps delivery (n, %)	6 (2.50)	58 (1.88)	0.501
NT MoM (median, IQR)	0.95 (0.80~1.13)	0.98 (0.83~1.17)	0.105
β-HCG MoM (median, IQR)	0.98 (0.60~1.62)	1.01 (0.63~1.62)	0.430
PAPP-A MoM (median, IQR)	0.91 (0.68~1.27)	0.99 (0.73~1.37)	0.001
AFP MoM (median, IQR)	2.97 (2.67~3.53)	1.03 (0.76~1.58)	0.000

*AFP* Alpha-fetoprotein, *β-HCG* β-human chorionic gonadotropin, *PAPP-A* Pregnancy associated plasma protein-A, *NT* Nuchal translucency, *MoM* Multiple of the median, *IQR* Interquartile range

A total of 594 pregnancies resulted in APOs, among which 181 in preterm birth, 32 in stillbirth, 81 in preeclampsia, and 362 in SGA. The incidence rate of overall APOs among women with MS-AFP ≥ 2.5 and < 2.5 MoM were 29.58 vs. 16.95% (*P* = 0.000). The incidence rate of each APO in the study population and its corresponding rate in the elevated and normal MS-AFP groups were 5.44% (11.67 vs. 4.96%, *P* = 0.000) for preterm birth, 0.96% (1.25 vs. 0.94%, *P* = 0.500) for stillbirth, 2.44% (6.25 vs. 2.14%, *P* = 0.001) for preeclampsia, and 10.89% (17.92 vs. 10.34%, *P* = 0.001) for SGA. Women with MS-AFP ≥ 2.5 MoM had increased risks (OR, 95% CI) of preterm birth (2.53, 1.65~3.88), preeclampsia (3.05, 1.71~5.43) and SGA (1.90, 1.34~2.69), while the risk of stillbirth was not significantly increased (1.33, 0.40~4.41) (Table 2).

**Table 2:** The association of first-trimester MS-AFP with adverse pregnancy outcomes

	MS-AFP ≥ 2.5 MoM (n, %)	MS-AFP < 2.5 MoM (n, %)	OR	95% CI	*P* value
Preterm birth	28 (11.67)	153 (4.96)	2.53	1.65 ~ 3.88	0.000
Stillbirth	3 (1.25)	29 (0.94)	1.33	0.40 ~ 4.41	0.500
Preeclampsia	15 (6.25)	66 (2.14)	3.05	1.71 ~ 5.43	0.001
SGA	43 (17.92)	319 (10.34)	1.90	1.34 ~ 2.69	0.001
Overall APOs	71 (29.58)	523 (16.95)	2.06	1.54 ~ 2.76	0.000

*OR* Odds ratio, *CI* Confidence interval, *SGA* Small for gestational age, *APOs* Adverse pregnancy outcomes

**Fig. 2:**
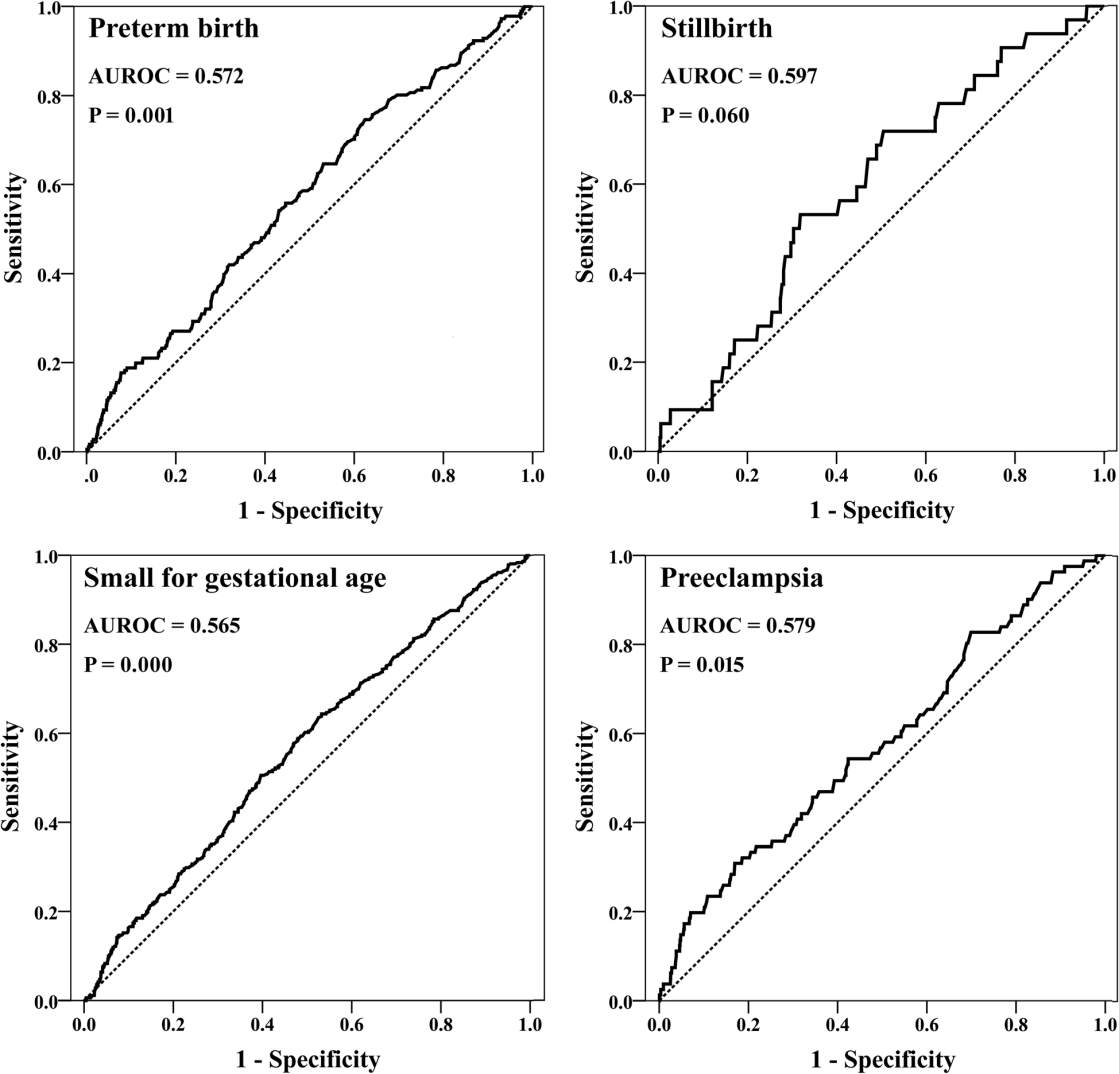
The ROC curves used to evaluate the predictive efficiencies of MS-AFP to preterm birth, stillbirth, preeclampsia and small for gestational age. The area under ROC curve was small (< 0.60) for the four APOs, and the significance was not obtained for stillbirth probably due to its lower incidence than that of the other three APOs. AUROC: the area under ROC curve

In order to figure out the association of individual MS-AFP level with the incidences of APOs, the ROC curve was used to evaluate the predictive efficiency. The areas under ROC curves (AUROC) were 0.569 for overall APOs (*P* = 0.000), 0.572 for preterm birth (*P* = 0.001), 0.597 for stillbirth (*P* = 0.060), 0.579 for preeclampsia (*P* = 0.015) and 0.565 for SGA (*P* = 0.000) (Fig. 2). It could be seen that the AUROC was small for each APO, and the significance was not obtained for stillbirth probably due to its lower incidence than that of the other three APOs.

Considering that MS-AFP might perform better for more severe APOs such as SGA < 3rd percentile, or preterm birth at < 32 weeks, or severe preeclampsia, we further evaluated the performances of MS-AFP for these subdivisions of APOs. It could be seen that MS-AFP did not perform remarkably better as predictors of more severe APOs (Table 3). When preterm birth was divided into spontaneous (*n* = 145) and iatrogenic (*n* = 36), a mildly improved AUROC was obtained: 0.631 for iatrogenic (*P* = 0.007) vs. 0.556 for spontaneous (*P* = 0.023) (Table 3). Since SGA consists also of group of healthy small fetuses, we tried to use ultrasound definition that was put forward by Gordijn et al. in 2016 to discriminate fetal growth restriction (FGR) cases [21]. One hundred four cases of FGR were defined in 362 SGA, and the performance of MS-AFP for FGR was mildly improved, with an AUROC of 0.602 (*P* = 0.000) compared to 0.565 for SGA (*P* = 0.000) (Table 3). To sum up, first-trimester MS-AFP was not a good predictor for these APOs, even if the subdivisions of APOs were applied for analysis.

**Table 3:** The performance of MS-AFP for the subdivisions of adverse pregnancy outcomes

APOs	n	AUROC	*P* value
Total preeclampsia	81	0.579	0.015
Severe preeclampsia	36	0.585	0.079
Total preterm birth	181	0.572	0.001
Preterm birth at < 32 weeks	19	0.551	0.456
Spontaneous preterm birth	145	0.556	0.023
Iatrogenic preterm birth	36	0.631	0.007
Total SGA (< 10th percentile)	362	0.565	0.000
Severe SGA (< 3rd percentile)	89	0.604	0.001
FGR	104	0.602	0.000

*APOs* Adverse pregnancy outcomes, *SGA* Small for gestational age, *FGR* Fetal growth restriction by ultrasound definition put forward by Gordijn et al. [21], *AUROC* Area under ROC curve

**Fig. 3:**
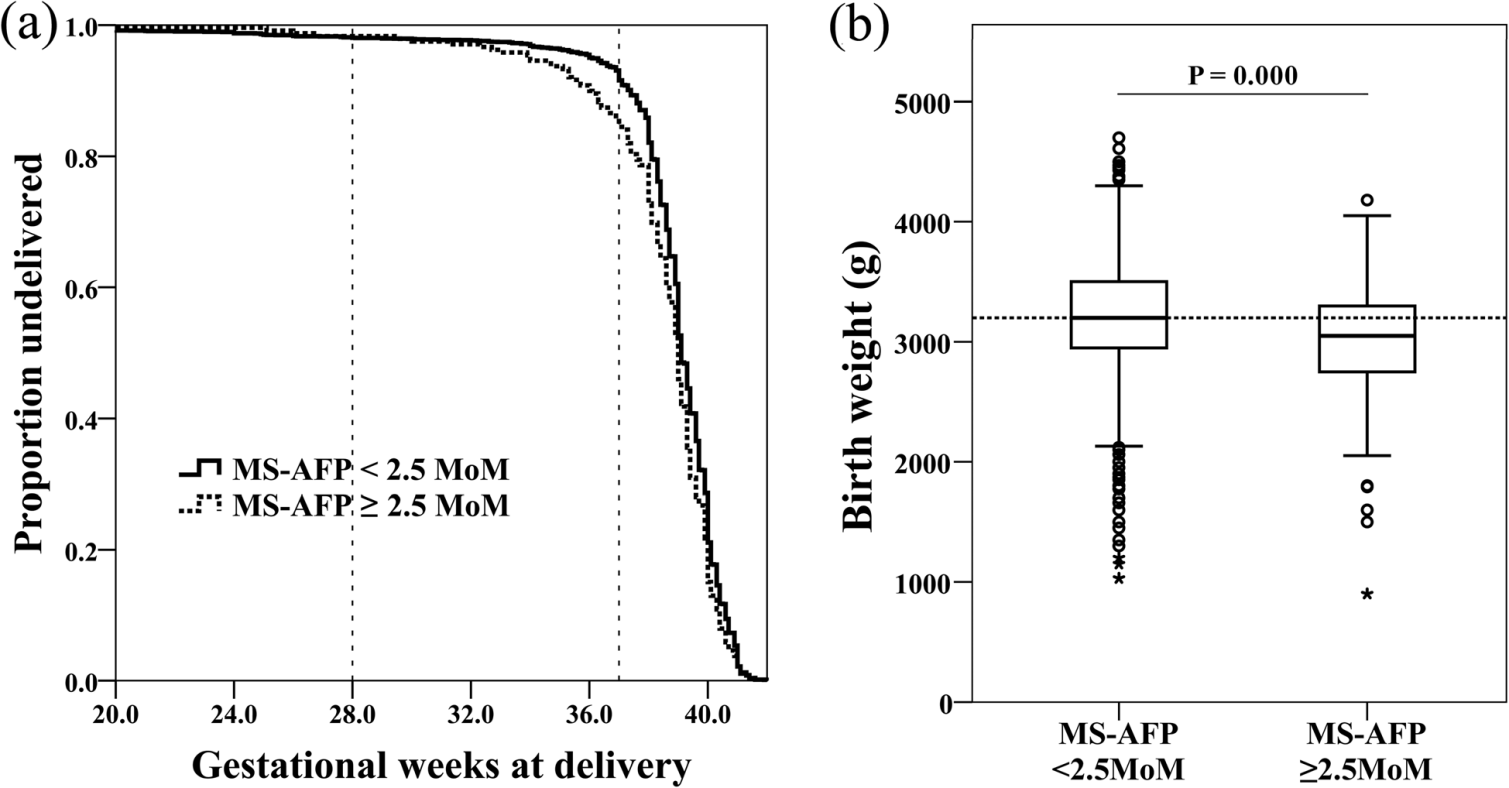
**a** The Kaplan-Meier curve used to record the gestational age at delivery for all participants. Women with MS-AFP ≥ 2.5 MoM had an earlier overall distribution of gestational weeks at delivery (Log-Rank test *P* = 0.004), but the overall difference was minuscule. **b** A boxplot showing the birth weights of all neonates. Women with MS-AFP ≥ 2.5 MoM had a lower overall distribution of neonatal birth weights (Median test *P* = 0.000), but the actual difference was minuscule: the medians of the two groups were 3200 and 3050 g respectively

To concretely show the discriminatory power of MS-AFP for preterm birth, a Kaplan-Meier curve was used to record the gestational weeks at delivery for all participants. Women with MS-AFP ≥ 2.5 MoM had an earlier overall distribution of gestational weeks at delivery (*P* = 0.004). However, the actual difference was minuscule; the most significant difference in gestational weeks at delivery were concentrated in 36~37 weeks in which the fetuses are close to maturity and are less prone to adverse outcomes (Fig. 3a). A boxplot was used to show the birth weights of all neonates. Women with MS-AFP ≥ 2.5 MoM had a lower overall distribution of neonatal birth weights (*P* = 0.000). However, the actual difference was also minuscule; the medians of neonatal birth weight were 3200 and 3050 g respectively in the two groups (Fig. 3b).

## Discussion

In this study, we found that elevated first-trimester MS-AFP is associated with increased risks of preterm birth, preeclampsia and SGA. However, the predictive efficiencies were low and it is not a good predictor for these APOs.

In our previous studies on second-trimester MS-AFP and APOs, the AUROC was 0.686, 0.717 and 0.611 for preterm birth, preeclampsia and SGA, respectively; women with second-trimester MS-AFP ≥ 2.5 MoM had increased risks (OR, 95% CI) of preterm birth (4.10, 2.44~6.88), preeclampsia (3.95, 2.23~6.99) and SGA (3.45, 1.91~6.21) [22]. The results were in general agreement with other studies on second-trimester MS-AFP and APOs [6, 9–12]. A case-control study on MS-AFP and preeclampsia also revealed that MS-AFP was elevated in both the first and the second trimesters in pregnancies that developed preeclampsia, but the performance of second-trimester MS-AFP combined with maternal factors for preeclampsia screening was better than that of first-trimester MS-AFP [23]. Taking others’ and our findings together, we can conclude that first-trimester MS-AFP is less predictive of APOs than second-trimester MS-AFP.

The lower efficiencies of first-trimester MS-AFP than second-trimester MS-AFP for the predictions of APOs may be interpreted by the mechanism of MS-AFP elevation. Unlike other maternal serum markers produced by the placenta, AFP is fetal-derived during pregnancy. Elevated MS-AFP is thought to reflect excessive placental permeability that leads to the escape of AFP from the fetus to the mother [24, 25]. This was verified in the animal studies that lipopolysaccharide-induced intrauterine inflammation gave rise to increased placental permeability and lipopolysaccharide-treated pregnant rats had elevated MS-AFP [26, 27]. It has been known that the placenta plays important roles in the pathophysiology of a number of APOs. Preterm birth, preeclampsia and SGA are currently considered as “placenta-mediated” disorders [28–30]. A series of evidences indicated that increased placental permeability might occur in these disorders [31–33]. Therefore, we may infer that, starting from the end of the first trimester when the primitive placenta undergoes remodeling to form the definitive organ [34], the increase in placental permeability is a gradually developed process under certain pathological conditions. In the process, placental permeability to AFP is also increasing from the first to the second trimester. As a typical placenta-mediated disease, the pathogenesis of preeclampsia may support our inference by its “two-stage” model: maternal vascular malperfusion caused by poor trophoblast uterine invasion and impaired transformation of the spiral arteries in the first trimester leads to placental inflammation and vascular endothelial injury that cause placental dysfunction and precipitate the onset of the maternal syndrome in the later gestation [35].

In the recent years, much concern has been raised about the first-trimester screenings for APOs which are considered as a window of opportunity to predict and prevent these disorders [13]. In addition to preeclampsia, it has been reported that low-dose aspirin use during pregnancy is also effective in the prevention of other APOs [36–38]. ACOG Committee opinion recommends that low-dose aspirin prophylaxis commenced optimally before 16 gestational weeks [4]. Given this, the need for early predictions of APOs is important. Although our findings indicated that first-trimester MS-AFP is not efficient enough for the predictions of APOs, it showed significant associations with these disorders and may play a role in the combined multi-marker screenings. In addition to preeclampsia screening which has achieved much progress, the first-trimester combined multi-marker screenings for other APOs have also been explored in the recent years. Beta et al. described a model based on the factors identifiable in the first trimester which detected 18.4 and 38.2% of preterm birth for nulliparous or primiparous women at a 10% false-positive rate [39]. Greco et al. described a screening method combining cervical echography with maternal characteristics and obstetric history which detected 54.8% of preterm birth at a 10% false-positive rate [40]. Poon et al. published a predictive algorithm involving demographic, biophysical and biochemical parameters which had a detection rate of 55.5% for preterm SGA and 44.3% for term SGA with a 10.9% false-positive rate [41]. Akolekar et al. reported a screening model base on maternal factors, PlGF, fetal ductus venosus and uterine artery pulsatility index which predicted 42% of all stillbirths and 61% of those due to impaired placentation at a false-positive rate of 10% [42]. These models for APOs screenings are in the developing stage. Incorporation of further variables may help to improve the screening efficacies, and first-trimester MS-AFP may be a promising biochemical marker.

It was reported that singleton pregnancies following assisted reproductive technology had increased risk of APOs; MS-AFP levels in women who were conceived using assisted reproductive technology was different from that of natural conceptions [43–45]. These might be the reason why elevated MS-AFP group had higher proportion of assisted reproductive technology in our study. Besides, elevated MS-AFP group had lower maternal serum PAPP-A. The possible reason is that PAPP-A is also a marker for APOs, and decreased PAPP-A is associated with increased risk of APOs [46]. In our data analysis, it was indeed that MS-AFP was negatively correlated to PAPP-A (Pearson correlation *P* = 0.010). In China, the caesarean section rate increased from 29% in 2008 to 35% in 2014. After the relaxation of the one-child policy in November 2013, considering the safety of delivery, hospitals in China were generally reluctant to try vaginal birth after cesarean (VBAC). In 2016, the caesarean section rate was 41.1% in China [47]. That was the reason why we had a high caesarean section percentage in this study.

## Conclusions

This study provides evidence that elevated first-trimester MS-AFP is associated with increased risk of preterm birth, preeclampsia and SGA, but the predictive efficiencies were low for these APOs, even if the subdivisions of APOs were applied for analysis. However, as the importance of first-trimester combined multi-marker screenings for APOs are increasingly emphasized in the recent years, first-trimester MS-AFP may also be a useful marker in the future studies on this subject.

## Data Availability

The datasets used and analyzed during the current study are available from the corresponding author on reasonable request.

## Abbreviations

APOs: Adverse pregnancy outcomes
AUROC: Area under receiver operating characteristic curve
CI: Confidence interval
FGR: Fetal growth restriction
MoM: Multiple of the median
MS-AFP: Maternal serum alpha-fetoprotein
NT: Nuchal translucency
ONTDs: Open neural tube defects
OR: Odds ratio
PAPP-A: Pregnancy-associated plasma protein-A
ROC: Receiver operating characteristic
SGA: Small for gestational age
β-HCG: β-human chorionic gonadotropin
